# Introduction pathway and climate trump ecology and life history as predictors of establishment success in alien frogs and toads

**DOI:** 10.1002/ece3.261

**Published:** 2012-07

**Authors:** Alfredo Rago, Geoffrey M While, Tobias Uller

**Affiliations:** Department of Zoology, Edward Grey Institute, University of OxfordOxford, OX13PS, United Kingdom

**Keywords:** Amphibians, colonization, extinction, invasion, life history, range expansion

## Abstract

A major goal for ecology and evolution is to understand how abiotic and biotic factors shape patterns of biological diversity. Here, we show that variation in establishment success of nonnative frogs and toads is primarily explained by variation in introduction pathways and climatic similarity between the native range and introduction locality, with minor contributions from phylogeny, species ecology, and life history. This finding contrasts with recent evidence that particular species characteristics promote evolutionary range expansion and reduce the probability of extinction in native populations of amphibians, emphasizing how different mechanisms may shape species distributions on different temporal and spatial scales. We suggest that contemporary changes in the distribution of amphibians will be primarily determined by human-mediated extinctions and movement of species within climatic envelopes, and less by species-typical traits.

## Introduction

Understanding the processes behind the past, present, and future patterns of biodiversity is fundamental to ecology and evolutionary biology. Human activities are now the dominant force shaping species distributions, partly by dramatically increasing the rate at which species encounter novel environments (Tatem 2007, 2009). It is therefore important that we study the processes underlying the establishment of alien species, not only because they may pose significant biological and economical threats (Elton 1958; Gewin 2005; Pimentel et al. 2005) but also because establishment of breeding populations is an important step during geographic range expansion, which strongly contributes to the potential for further ecological and evolutionary diversification (reviewed in Sax et al. 2005; Cadotte et al. 2006). Furthermore, introductions of nonnative species provide an opportunity to address whether the factors that promote human-mediated range expansion are similar to those that determine species ranges on different temporal (e.g., over evolutionary time) and spatial (e.g., local extinctions within the native range) scales.

The factors that contribute to the establishment success of nonnative species can be classified into three different categories (Blackburn et al. 2009a). Event-level factors comprise properties of the release such as the number of individuals introduced, the number of releases and the timing of the introductions. Such factors have been shown to have a major effect on establishment success in several taxa (Cassey et al. 2004; Colautti et al. 2006; Duggan et al. 2006; Dehnen-Schmutz et al. 2007; Lockwood et al. 2009). Properties of the introduced location (location-level traits) should also be relevant for establishment success, in particular the degree of abiotic and biotic similarity to the species’ native range (Duncan et al. 2001; Hayes and Barry 2008; Blackburn et al. 2009a). Finally, species traits may also facilitate persistence of alien species. For example, studies of bird introductions suggest that species with a broader habitat use are more likely to establish breeding populations outside of their native range (Cassey et al. 2004; Sol et al. 2005; Blackburn et al. 2009b). It is also arguable that species with traits that promote fast demographic growth rates (e.g., large clutch size) should be more likely to persist since those traits reduce the risk of extinction due to environmental and demographic stochasticity. Although evidence for the effect of each of these factors exist, their relative importance on a global scale is poorly understood as studies tend to focus on only a subset of factors, often are restricted to specific geographic locations, or fail to account for phylogenetic independence (reviews in Sol et al. 2007; Blackburn et al. 2009a, b).

Amphibians are interesting to study from a colonization perspective because of their high sensitivity to climate change (e.g., Parmesan 2006). While this leads us to expect that climatic similarity should be the most important factor for establishment success, recent evidence suggest that species characteristics can contribute to range shifts on both eco-logical and evolutionary time scales. For example, global range expansion of some anuran amphibians during the Oligocene was facilitated by evolutionary accumulation of a particular suite of phenotypic characteristics, including large body and clutch size and the presence of a free-living tadpole stage (van Bocxlaer et al. 2010). This raises intriguing questions regarding whether traits that promote evolutionary range expansion also contribute to establishment success on ecological time scales. Recent studies of amphibian declines have also identified a set of species characters that are associated with extinction risk (Cooper et al. 2008; Sodhi et al. 2008). These partly overlap with the traits that promote evolutionary range expansion but also include more composite characters such as the degree of habitat specialization (Sodhi et al. 2008; van Bocxlaer et al. 2010). These traits could also make small populations less likely to go extinct during colonization since they should reduce the impact of stochastic factors on population growth. Indeed, introduced populations are similar to endangered ones in several respects since both are characterized by a small number of individuals, a fragmented and restricted range and high local extinction probabilities. As such, it has been proposed that successful establishment and local extinction might be considered opposite processes (e.g., Lockwood 1999; but see Jeschke 2008; Blackburn and Jeschke 2009).

Here, we compare the impact of a comprehensive suite of event, location, and species-level factors on establishment success of amphibian introductions. We relate our results to previous works that have identified factors that promote range expansion and reduce extinction risk in amphibians (and other vertebrates) to test whether the factors that promote evolutionary range expansion and make a species less vulnerable to extinction also make it a successful colonizer on ecological time scales.

## Methods

### Invasion database

We collected data on establishment success from the recently published summary of all known amphibian introductions by Kraus (2009). We decided to exclude from our analysis the orders Caudata and Gymnophiona due to the limited data for these groups, problems associated with unambiguously comparing traits (such as body size) between these taxa and Anurans, and to allow more realistic comparison with a recent study that focused on evolutionary range expansion in a subset of Anurans (van Bocxlear et al. 2010). The locations reported include countries, islands, or United States of America. Therefore, we used species-jurisdiction pairs as the unit for our study, including only events with known outcome (success or failure) and fully identified species. Since the summary we used is at the scale of whole jurisdictions, we report a number of introductions to regions that host native populations of the species. These entries are to be interpreted as introductions of the species into a nonnative location within a jurisdiction that also hosts native populations of that species. We also note that the introduction record (and thus our dataset) is skewed toward successful attempts, especially in the case of unintentional releases. This is unfortunately a universal problem in the comparative study of invasive species, but one that needs to be kept in mind when interpreting the results.

While persistence time and number of releases are consistently reported as important variables in the literature, we were unable to assess them in this study due to the high number of missing or inconsistent records (see Data Analysis). To provide an estimate of those parameters, we reported whether any of the introduction attempts were intentional, as those are most often associated with larger number of individuals released, more release events, and a benign timing and small-scale location of the introductions (Kraus 2009). Species-jurisdiction pairs with at least one introduction reported as biocontrol, food, experimental, or intentional release were classified as intentional release. Pairs for which only unintentional introduction attempts are reported (cargo stowaway, pet trade, nursery trade, and aquaculture contaminant) were classified as unintentional release. Remaining events were classified as unknown intentionality.

### Climate matching

For each species-jurisdiction pair, we assessed the climatic similarity between the native and introduced range by employing an environmental distance approach. This is preferable from using simple proxies of climate, such as latitude (e.g., Tingley et al. 2011) as it directly measures the difference in the relevant environmental parameters. We choose to use the DOMAIN algorithm (Carpenter et al. 1993) as implemented in DIVAGIS version 7.4 (Hijmans et al. 2001). This algorithm, originally designed to predict species’ distributions, provides a robust method to compare bioclimatic parameters between a species’ native habitat and other regions by means of a two-step process. It firstly records the climatic parameters of areas with reported species occurrences and uses them to model a bioclimatic envelope for the species. It then measures the distance between this envelope and the introduced range on a cell-by-cell basis. We used the proportion of cells of the introduced range that scored above the 90th percentile of distance of the species' climate envelope as our metric for estimating climate matching. While other quantiles (the 50th, 80th, and 95th) generated qualitatively similar results (with introductions remaining either as high or low scoring), we choose the 90th due to its higher proportion of intermediate values, which grants a greater resolution for the analysis.

Global climatic data with 2.5 min (latitude × longitude) resolution was obtained from the WorldClim database. The 14 bioclimatic parameters used in our analysis are the same as those used by Bomford et al. (2008) and are listed in the Supporting information (Table S1). Species occurrences were downloaded from the GBIF database version 1.3 (Guralnick et al. 2007; Yesson et al. 2007) using openModeller Desktop version 1 (de Souza Muńoz et al. 2011), excluding species with fewer than six occurrences. Since the GBIF database also comprises introduced populations, we expected a systematic positive bias of climate-matching scores toward successful introduction events. Although the DOMAIN algorithm is robust to the presence of outliers (Carpenter et al. 1993), we corrected for this bias by excluding the cells with a perfect (100%) climate-matching score, which result from known occurrence data. Any bias should thus lead toward the conservative hypothesis of a positive effect of climate matching on establishment success. We also included the geographic distance between the native and introduced ranges, which could affect both the probability of unintentional introductions and the number and condition of introduced animals. The distance was calculated as the great circle distance (in km) from the centroids of the two regions, log transformed and normalized to the final dataset. To estimate the effect of congeneric species presence (Duncan and Williams 2002; Tingley et al. 2011), we collected data on the presence/absence as well as number of native congeners for each species-jurisdiction pair by using the geographic and taxonomic data available from the IUCN redlist (http://wwwiucnredlist.org/initiatives/ampibians). The genus of each introduced species was entered into the database and the results examined for overlap with the introduction location.

### Life-history and species traits

We gathered data on life-history traits from online resources (amphibiaweb and IUCN redlist), primary literature, and field guides. We chose traits for which there are theoretical reasons to expect an impact on establishment success, or that previously have been shown to predict extinction risk and range expansion (e.g., Sodhi et al. 2008; Cooper et al. 2008; van Bocxlaer et al. 2010). Species traits included in our analysis comprise minimum body size at maturity (reported as snout-vent length in millimeters and log transformed), minimum clutch size (log transformed), presence of free-living aquatic larval stages (present/absent), geographic range size (reported in km^2^ and log transformed), and habitat breadth (log transformed). While there are several other species characters that have been shown to play a role in invasion success (e.g., brain size), data for those traits are missing for most of the species analyzed. We calculated native geographical range using the BerkeleyMapper interface for the IUCN aerials. We used the IUCN classification to assess the number of habitats inhabited by each species. We also reported their ability to inhabit terrestrial, seasonal, and artificial habitats following van Boxclaer et al. (2010). All online resources were accessed between February and March 2011.

### Data analysis

Invasion success was modeled using generalized linear mixed models (GLMMs) with establishment success (successful vs. unsuccessful) as the response variable, a binomial error structure, and a logit link function. We removed all entries with missing data before the analysis to avoid bias from different sizes of the dataset. All numeric variables were normalized to a mean of 0 and a standard deviation of 0.5 before analysis (following Grueber et al. 2011). Since the amphibian phylogeny is still poorly resolved, we used family, genus, and species (as per Frost 2011) as three nested random factors to control for phylogenetic nonindependence (Sol et al. 2007). We included the introduction location as a second random error structure to account for the correlation of introductions in the same area.

We initially included the reported number of introductions, coded either as a direct numeric variable or as a binary factor (single/multiple). However, neither contributed significantly to the models’ predictive power (data not shown), possibly due to the inaccurate and often missing record of introduction events, and were therefore omitted from our final model sets. Similarly, we resorted to use presence/absence data on congenerics rather than the number of species present since the latter did not provide a significant increase in the model's predictive power (data not shown). Furthermore, while we initially set out to evaluate the ability of species to live across a range of habitats (e.g., terrestrial, seasonal, and artificial), the subset of introduced species did not show sufficient variation to assess the importance of this factor since almost every introduced species occurs across all three habitat types. All of the variables analyzed were checked for collinearity. We found only two problematic correlations, with a strong correlation between body and clutch size and both being larger for species with tadpole stages. Because of this and also because data for body size and clutch size are missing for several of the introduced species, we generated a second dataset which excluded those variables. Our final dataset with body size and clutch size comprised 385 species-jurisdiction pairs and included 85 species from 37 genera. The second dataset instead comprised 408 pairs and included 99 species from 42 genera. Both datasets are provided in the Supporting information (Tables S2 and S3).

Models with different combinations of factors were scored according to second-order Akaike information criteria (AICc), which allows quantitative comparison of the predictive power of several nested models, each representing a separate hypothesis for which factors that are relevant for predicting establishment success (Burnham and Anderson 2002). For a more comprehensive exposition of the methods used, see Greueber et al. 2011. All statistical analyses were performed in R (R Development Core Team 2011) using the packages lme4 (Bates et al. 2011) for the generation of GLMMs, geosphere (Hijmans et al. 2011) for measuring the geographic distances, and MuMIn (Bartoń 2011) for the generation of model sets and model-averaged estimates. Graphics were generated using the ggplot package (Wickham 2009).

## Results

**Figure 1:**
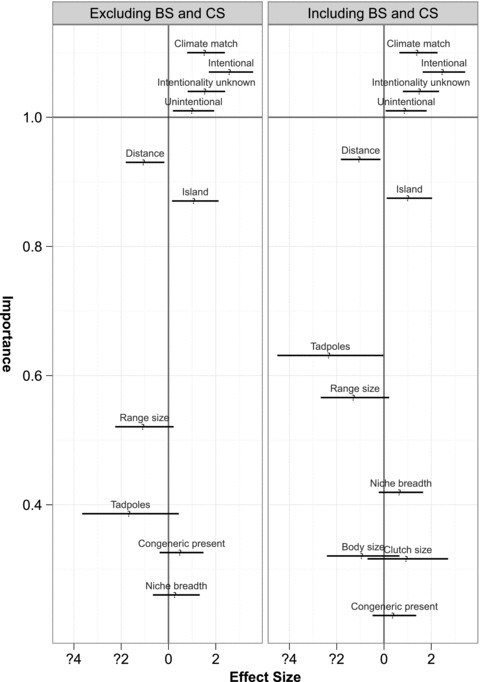
Average effect sizes (represented by dots) with 95% confidence intervals (represented by bars). Results from the analysis of the dataset including body size and clutch size are shown in the right panel. Results from the dataset excluding body size and clutch size are shown in the left panel. All factors are ordered vertically according to their relative importance. Factors included in all top models (intentionality and climate match) with a maximum importance of 1 are included at the top. Levels of categorical variables have been adjusted not to overlap.

The best scoring models for each dataset are shown in Tables 1 and 2. Since there is no clear best model but rather a set of top models with similar scores, we calculated the effect sizes of individual factors using model averaging, which generates estimates from the whole model set, weighting the individual models according to their relative predictive power and accounting for uncertainty in model selection (Burnham and Anderson 2002). The top model set used for model averaging includes all models that differ from the top model by less than 4 points of AICc. The effect sizes and the relative importance of the factors analyzed are shown in Figure 1 (with 95% confidence intervals) and reported in Table S1.

**Table 1:** Top 10 scoring models for the dataset including body size and clutch size, with the null model for the set at the bottom of the table. Variables are coded as follows: (1) minimum body size, (2) presence of congeneric species, (3) minimum clutch size, (4) distance from native range, (5) habitat breadth, (6) intentionality of release, (7) island versus mainland, (8) climate matching, (9) range size, (10) presence of larval stage

Factors	Delta AICc
4 + 6 + 7 + 8 + 10	0
4 + 6 + 7 + 8 + 9	1.1658
4 + 5 + 6 + 7 + 8 + 9	1.2094
4 + 5 + 6 + 7 + 8 + 9 + 10	1.2129
4 + 5 + 6 + 7 + 8 + 10	1.2226
3 + 4 + 6 + 7 + 8 + 10	1.2412
4 + 6 + 7 + 8 + 9 + 10	1.4550
2 + 4 + 6 + 7 + 8 + 10	1.5907
4 + 6 + 7 + 8 + 10	1.8732
4 + 6 + 7 + 8 + 9	1.9438
Intercept only	36.662

**Table 2:** Top 10 scoring models for the dataset excluding body size and clutch size, with the null model for the set at the bottom of the table. Variables are coded as follows: (1) minimum body size, (2) presence of congeneric species, (3) minimum clutch size, (4) distance from native range, (5) habitat breadth, (6) intentionality of release, (7) island versus mainland, (8) climate matching, (9) range size, (10) presence of larval stage

Factors	Delta AICc
4 + 6 + 7 + 8 + 9	0
4 + 6 + 7 + 8 + 10	0.1912
4 + 6 + 7 + 8	0.5389
2 + 4 + 6 + 7 + 8 + 9	1.1077
4 + 5 + 6 + 7 + 8	1.2222
2 + 4 + 6 + 7 + 8 + 9	1.3693
4 + 6 + 7 + 8 + 10	1.4790
2 + 4 + 6 + 7 + 8 + 10	1.5754
4 + 5 + 6 + 7 + 8 + 10	2.0674
4 + 6 + 8 + 10	2.2898
Intercept only	36.613

**Figure 2:**
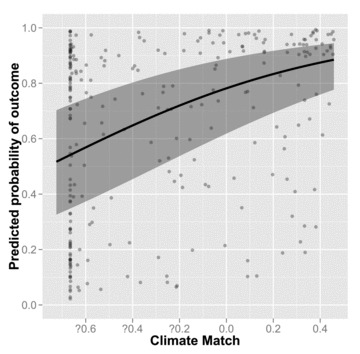
Predicted probability of establishment of alien amphibians as a function of the climatic similarity between the native range and the introduction location (black line) with 95% confidence intervals (gray area). Predicted establishment success of the species in the dataset is shown as dots. Predictions for this figure were based on the top-scoring model of the dataset excluding body size and clutch size.

The residual variance of the best model in each model set is reported in Table 3. Of the variables analyzed, two were present in all top-scoring models, with an effective relative importance of 1 and narrow confidence intervals that do not cross zero. Those are climate matching and intentionality of the introduction. In accordance with previous studies on amphibians and other taxa, the climatic similarity between the native range and the introduction location was one of the most important drivers of establishment success (Fig. 2). The pathways of introduction (intentional vs. unintentional) also strongly predicted establishment success, with intentional introductions being more likely to succeed than unintentional introductions in both of our datasets. The unknown category of introduction pathway was intermediate between the two.

**Table 3:** Residual variance explained by the error structures in models with and without body size (BS) and clutch size (CS)

Groups	Variance	Standard deviation	Dataset
Introduction location	2.833	1.683	Including BS and CS
Species	0.302	0.550	Including BS and CS
Genus	0.184	0.429	Including BS and CS
Family	0.161	0.402	Including BS and CS
Introduction location	3.073	1.753	Excluding BS and CS
Species	0.086	0.293	Excluding BS and CS
Genus	0.414	0.644	Excluding BS and CS
Family	0.420	0.648	Excluding BS and CS

Another strong predictor of establishment success was whether the introduction was to an island or a continent, with introductions to islands being more likely to be successful. This factor remained equally important when controlling for presence of congeneric species in the introduced location and distance between the native and introduced ranges. Distance itself is also supported as negatively affecting establishment success, despite a relatively small effect size. While not as strongly supported as climate matching and intentionality, both factors have high relative importance and confidence intervals that do not include zero (Fig. 1).

Of the five species-level traits analyzed, only one (presence of aquatic larval stages) shows an effect on establishment success, but is supported only in the dataset that includes entries complete for both body and clutch size (and thus represents a smaller subset). Contrary to what has been reported for evolutionary range expansion of toads (van Bocxlaer et al. 2010), presence of tadpoles appears to have a negative effect on the probability of establishment. All of the other species factors (niche breadth, native range size, clutch size, body size) and congeneric presence received weak support, since they present both low relative importance values as well as wide confidence intervals that overlap with zero.

Introduction location accounted for a major component of the residual variance. On the other hand, no single taxonomic level was able to explain a major proportion of residual variance (Table 3). This suggests that, once the characteristics of the location and of the release event have been accounted for, none of the examined taxa is a better colonizer than others.

## Discussion

Successful range expansion is the outcome of a series of events from dispersal of species outside of their native range to successful reproduction and recruitment. Here, we focused on one particular aspect of this process; establishment success subsequent to human-mediated dispersal. Our comprehensive analysis of predictors of establishment success in frogs and toads shows that whether or not a species establishes a breeding population outside of its native range is primarily driven by event- and location-level factors. Our analysis highlights especially the prime importance of introduction pathways and climatic similarity between the native range and introduction locality, in contrast with the weak effects of species-specific characteristics.

Our study contributes to what we believe is an emerging consensus in the invasion literature that where, when, how, and how many individuals of a species are introduced are more important for establishment success than species characteristics (Cassey et al. 2004; Blackburn et al. 2009b). For example, although some phenotypic traits positively affect establishment success in birds (e.g., range size, diet, and habitat breadth; Blackburn et al. 2009b), their effect size is generally small compared to the effect of the number of individuals released and the number of releases (often referred to as propagule pressure) (Kolar and Lodge 2001; Cassey et al. 2004; Lockwood et al. 2005, 2009). Unfortunately, amphibian introductions rarely include accurate records on propagule pressure itself. Thus, it is unknown to what extent the strong positive effect of intentional introductions in our study reflects direct effects of propagule pressure versus other aspects of the introduction history, such as the timing and small-scale location of introduction (e.g., into suitable breeding ponds). For the time being, it seems safe to assume that both are important.

The introduction site is expected to have a strong impact on the probability of establishment in nonnative species. Based on the evidence for local climatic adaptation in amphibians (e.g., Laugen et al. 2003; Olsson and Uller 2003; Phillimore et al. 2010), we predicted that climatic similarity between the native range and the introduction locality would be the best individual predictor of establishment success. This was indeed the case, which corroborates results from previous analyses (Bomford et al. 2008). The overall effect of climate seems to be somewhat stronger than previously reported for birds (e.g., Duncan et al. 2001; summarized in Blackburn et al. 2009a), perhaps reflecting more pronounced local adaptation to climatic conditions in ectotherms than in endotherms. We also found consistent support for an increased establishment success for species introduced to islands, which may reflect differences in ecological conditions compared to the mainland. For example, because amphibians are generally quite poor dispersers across open sea (Duellman and Trueb 1986; Brown and Guttman 2002; but see Vences et al. 2003), competition on islands may be relatively minor compared to that on the mainland. Interestingly, no such pattern has been shown in birds, a taxon with higher rates of natural dispersal and generally relatively weak island effects (Cassey et al. 2004; Blackburn et al. 2009a). Our analysis also suggests that some previously reported location-level ecological effects on establishment success (e.g., a positive effect of congeneric presence; Tingley et al. 2011) are better explained by short geographic distance between the native and introduced location and climatic similarity. Nevertheless, the location error structure still explains an large amount of residual variance, which suggests the presence of more unaccounted factors that influence how suitable a region is for introduced species.

Morphological and life-history traits have been shown to be important for changes in amphibian distributions on both global and local scales. For example, a recent comparative study suggested that evolutionary range expansion in toads was associated with particular phenotypic traits, including large body size, large clutch size, and a free-living tadpole stage (van Bocxlaer et al. 2010). If the same traits would predict range expansion on ecological time scales, it could point toward an important link between ecological and evolutionary processes. However, we found very little support for a positive effect of clutch size and body size on establishment success. Recent studies of extinction risk have also failed to find much support for a direct effect of body size or reproductive output (Cooper et al. 2008; Sodhi et al. 2008), suggesting that these traits are relatively unimportant for predicting the fate of contemporary amphibian populations.

A possible exception is the presence of free-living acquatic larval stages, which decreased the chances of establishment success. Intriguingly, this finding mirrors the positive effect of direct development in preventing amphibian declines (Sodhi et al. 2008). While the sample of species with direct development in our dataset is highly nonrandom both with respect to taxonomy (58 out of 60 records are for the Eleutherodactylidae) and aspects of their introduction (e.g., they have commonly been introduced to islands), the effect remained when controlling for those variables. Thus, it is possible that direct development is advantageous in at least some circumstances, for example, by making establishment success less dependent on the presence of suitable water bodies for breeding. A similar argument could be made for the degree of ecological specialization. Indeed, a recent meta-analysis of bird introductions found that habitat breadth was the strongest predictor of establishment success among all species traits examined (Blackburn et al. 2009b). However, range size and habitat breadth were both poor predictors of establishment success in alien amphibians, possibly because introduced species (which is a nonrandom subset of amphibians; Tingley et al. 2010) are often able to find suitable breeding habitats in a diversity of ecological conditions even if they are relatively specialized (e.g., only breeding in permanent bodies of water). Furthermore, perhaps a more important difference between species with tadpoles and those with direct development is that the latter have decreased demographic variance (Green 2003), an effect that might reduce the extinction risk from random fluctuations in population size during the establishment phase. If so, the only species character that might affect establishment success in our study does so because it reduces variation in population size rather than by enabling adaptation to a broader range of ecological conditions.

Overall, these results clearly show that characters that make a species vulnerable to extinction do not necessarily make it a poor colonizer (Jeschke 2008; Blackburn and Jeschke 2009). The lack of a relationship between establishment success and extinction risk is also supported by the absence of a strong phylogenetic signal in our analyses, which contrasts with the substantial phylogenetic effects on extinction risk (Corey and Waite 2008). This could have important implications for predicting changes in species distributions since it suggests that species with relatively high extinction rates in their native range may nevertheless persist via human-mediated dispersal. Furthermore, our results reveal that the species traits involved in anuran range expansion on an evolutionary timescale (van Bocxlaer et al. 2010) do not contribute to establishment success in modern times. However, it should be noted that species that inhabit a wide range of habitats will have a wider climatic envelope and thus a higher average climate-matching score. Since the colonization of diverse habitats is the result of dispersal history and species traits that promote local adaptation or phenotypic plasticity, it is possible that the positive effect of climate matching in studies like ours would be better explained by one or several adaptations shared by species living in a broad suite of environments. Furthermore, it is important to emphasize that those species characteristics that are unimportant for establishment success might be fundamental for enabling dispersal or introduction, and therefore might influence patterns of range expansion even if there is no bias in the establishment stage of colonization. Indeed, there is a substantial taxonomic bias toward northern hemisphere, large-bodied species of vertebrates with wide geographic ranges being more likely to be introduced (e.g., Blackburn and Duncan 2001; Sol 2007), a pattern that holds also among amphibians (Tingley et al. 2010). As human-mediated transportation and release becomes the primary cause of introductions outside of the species’ native ranges, traits that promote natural dispersal may become less important in shaping distributions than those that increase human-induced introduction probability and survival in a wider range of environments.

In summary, we have shown that establishment success of introduced amphibians is mainly driven by the pathway of introduction, introduction locality, and favorable climatic conditions. There was little support for a role of species characteristics or phylogeny, suggesting that changes in geographic ranges in amphibians during the Anthropocene (Steffen et al. 2011) will largely be determined by human-mediated extinctions and how humans influence dispersal within climatic zones.

## Supporting Information

**Table S1.** The 14 bioclimatic parameters used for the climate-matching analysis.

**Table S2.** Supplementary dataset for the analysis excluding body size and clutch size.

**Table S3.** Supplementary dataset for the analysis including body size and clutch size.

**Table S4.** The effect sizes, 95% lower and upper confidence intervals and relative importance of each factor for both datasets.

## References

[b1] Bartoń K (2011). MuMIn: multi-model inference. http://CRAN.R-project.org/package=MuMIn.

[b2] Bates D, Maechler M, Bolker B (2011). lme4: linear mixed-effects models using S4 classes. http://CRAN.R-project.org/package=lme4.

[b3] Blackburn TM, Duncan RP (2001). Establishment patterns of exotic birds are constrained by non random patterns of introduction. J. Biogeogr.

[b4] Blackburn TM, Jeschke JM (2009). Invasion success and threat status: two sides of a different coin?. Ecography.

[b5] Blackburn TM, Lockwood JL, Cassey P (2009a). Avian invasions: the ecology and evolution of exotic birds.

[b6] Blackburn TM, Cassey P, Lockwood JL (2009b). The role of species traits in the establishment success of exotic birds. Glob. Change Biol.

[b7] Bomford M, Kraus F, Barry SC, Lawrence E (2008). Predicting establishment success for alien reptiles and amphibians: a role for climate matching. Biol. Invasions.

[b9] Brown RM, Guttman SI (2002). Phylogenetic systematics of the *Rana signata* complex of Philippine and Bornean stream frogs: reconsideration of Huxley's modification of Wallace's Line at the Oriental-Australian faunal zone interface. Biol. J. Linn. Soc.

[b10] Burnham KP, Anderson DR (2002). Model selection and multimodel inference. A practical information-theoretic approach.

[b11] Cadotte MW, McMahon SM, Fukami T (2006). Conceptual ecology and invasion biology: reciprocal approaches to nature.

[b12] Carpenter G, Gillison AN, Winter J (1993). DOMAIN: a flexible modelling procedure for mapping potential distributions of plants and animals. Biodivers. Conserv.

[b13] Cassey P, Blackburn TM, Sol D, Duncan RP, Lockwood LJ (2004). Global patterns of introduction effort and establishment success in birds. Proc. R. Soc. Lond. B.

[b14] Colautti RI, Grigorovich IA, MacIsaac HJ (2006). Propagule pressure: a null model for biological invasions. Biol. Invasions.

[b15] Cooper N, Bielby J, Thomas GH, Purvis A (2008). Macroecology and extinction risk correlates of frogs. Glob. Ecol. Biogr.

[b16] Corey SJ, Waite TA (2008). Phylogenetic autocorrelation of extinction threat in globally imperilled amphibians. Divers. Distrib.

[b17] Dehnen-Schmutz K, Touza J, Perrings C, Williamson M (2007). The horticultural trade and ornamental plant invasions in Britain. Conserv. Biol.

[b18] de Souza Muńoz ME, Giovanni RD, de Siqueira MF, Sutton T, Brewer P, Pereira RS, Canhos DAL, Canhos VP (2011). openModeller: a genetic approach to species’ potential distribution modelling. Geoinformatica.

[b19] Duellman WE, Trueb L (1986). Biology and amphibians.

[b20] Duggan IC, Rixon CAM, MacIsaac HJ (2006). Popularity and propagule pressure: determinants of introduction and establishment of aquarium fish. Biol. Invasions.

[b21] Duncan RP, Williams PA (2002). Ecology—Darwin's naturalization hypothesis challenged. Nature.

[b22] Duncan RP, Bomford M, Forsyth DM, Conibear L (2001). High predictability in introduction outcomes and the geographical range size of introduced Australian birds: a role for climate. J. Anim. Ecol.

[b23] Elton CS (1958). The ecology of invasions by animals and plants.

[b24] Frost DR (2011). Amphibian species of the world: an online reference. http://research.amnh.org/vz/herpetology/amphibia/.

[b25] Gewin V (2005). Eco-defense against invasions. PLoS Biol.

[b26] Green D (2003). The ecology of extinction: population fluctuation and decline in amphibians. Biol. Conserv.

[b27] Grueber CE, Nakagawa S, Laws LJ, Jamieson IG (2011). Multimodel inference in ecology and evolution: challenges and solutions. J. Evol. Biol.

[b28] Guralnick RP, Hill AW, Lane M (2007). Towards a collaborative, global infrastructure for biodiversity assessment. Ecol. Lett.

[b29] Hayes KR, Barry SC (2008). Are there any consistent predictors of invasion success?. Biol. Invasions.

[b30] Hijmans RJ, Guarino L, Cruz M, Rojas E (2001). Computer tools for spatial analysis of plant genetic resources data: 1. DIVA-GIS. Plant Genet. Res. News.

[b31] Hijmans RJ, Williams E, Vennes C (2011). Geosphere: spherical trigonometry. http://CRAN.R-project.org/package=geosphere.

[b32] Jeschke JM (2008). Across islands and continents, mammals are more successful invaders than birds. Divers. Distrib.

[b33] Kolar CS, Lodge DM (2001). Progress in invasion biology: predicting invaders. Trends Ecol. Evol.

[b34] Kraus F (2009). Alien reptiles and amphibians: a scientific compendium and analysis.

[b35] Laugen AT, Laurila A, Räsanen K, Merilä J (2003). Latitudinal counter gradient variation in the common frog (*Rana temporaria*) development rates—evidence for local adaptation. J. Evol. Biol.

[b36] Lockwood JL (1999). Using taxonomy to predict success amongst introduced avifauna: relative importance of transport and establishment. Conserv. Biol.

[b37] Lockwood JL, Cassey P, Blackburn TM (2005). The role of propagule pressure in explaining species invasions. Trends Ecol. Evol.

[b38] Lockwood JL, Cassey P, Blackburn TM (2009). The more you introduce the more you get: the role of colonization pressure and propagule pressure in invasion ecology. Divers. Distrib.

[b39] Olsson M, Uller T (2003). Thermal environment, survival and local adaptation in the common frog, *Rana temporaria*. Evol. Ecol. Res.

[b40] Parmesan C (2006). Ecological and evolutionary responses to recent climate change. Ann. Rev. Ecol. Evol. Syst.

[b41] Phillimore AB, Hadfield JD, Jones OR, Smithers RJ (2010). Differences in spawning date between populations of common frog reveal local adaptation. Proc. Natl. Acad. Sci. USA.

[b42] Pimentel D (2005). Aquatic nuisance species in the New York State Canal and Hudson River systems and the Great Lakes Basin: an economic and environmental assessment. Environ. Manage.

[b43] Sax DF, Stachowicz JJ, Gaines SD (2005). Species invasions: insights into ecology, evolution, and biogeography.

[b44] Sodhi NS, Bickford D, Diesmos AC, Lee TM, Koh LP, Brook BW, Sekercioglu CH, Bradshaw CJA (2008). Measuring the meltdown: drivers of global amphibian extinction and decline. PLoS ONE.

[b45] Sol D, Nentwig W (2007). Do successful invaders exist? Pre-adaptations to novel environments in terrestrial vertebrates. Biological invasions.

[b46] Sol D, Duncan RP, Blackburn TM, Cassey P, Lefebvre L (2005). Big brains, enhanced cognition, and response of birds to novel environments. Proc. Natl. Acad. Sci. USA.

[b47] Sol D, Vilà M, Kühn I (2007). The comparative analysis of historical alien introductions. Biol. Invasions.

[b48] Steffen W, Jacques G, Crutzen P, McNeill J (2011). The Anthropocene: conceptual and historical perspective. Philos. Transac. R. Soc. Lond. B Biol. Sci.

[b49] Tatem AJ (2007). Climatic similarity and biological exchange in the worldwide airline transportation network. Philos. Transac. R. Soc. Lond. B Biol. Sci.

[b50] Tatem AJ (2009). The worldwide airline network and the dispersal of exotic species: 2007–2010. Ecography.

[b51] Tingley R, Romagosa CM, Kraus F, Bickford D, Phillips BL, Shine R (2010). The frog filter: amphibian introduction bias driven by taxonomy, body size and biogeography. Glob. Ecol. Biogeogr.

[b52] Tingley R, Phillips BL, Shine R (2011). Establishment success of introduced amphibians increases in the presence of congeneric species. Am. Nat.

[b53] van Bocxlaer I, Loader SP, Roelants K, Biju SD, Menegon M, Bossuyt F (2010). Gradual adaptation toward a range-expansion phenotype initiated the global radiation of toads. Science.

[b54] Vences M, Vieites DR, Glaw F, Brinkmann H, Kosuch J, Veith M, Meyer A (2003). Multiple overseas dispersal in amphibians. Proc. R. Soc. Lond. B Biol. Sci.

[b55] Wickham H (2009). ggplot2: elegant graphics for data analysis.

[b56] Yesson C, Brewer PW, Sutton T, Caithness N, Pahwa JS, Burgess M, Gary WA, White RJ, Jones AC, Bisby FW (2007). How global is the global biodiversity information facility?. PLOS ONE.

