# Supplementary material for: Introduction pathway and climate trump ecology and life history as predictors of establishment success in alien frogs and toads
Source: Ecol Evol. 2012 Jul;2(7):1437–45. doi: 10.1002/ece3.261 (PMC3434934; doi:10.1002/ece3.261)
Supplement: Supplementary file 1 [file ece30002-1437-SD1.doc]

**Table S1.** The 14 bio-climatic parameters used for the climate matching analysis.

| **Bioclimatic Parameter** |  |
| --- | --- |
| **Temperature parameters** | Minimum of coolest month |
|  | Maximum of warmest moth |
|  | Average range |
|  | Mean of coolest quarter |
|  | Mean of warmest quarter |
|  | Mean of wettest quarter |
|  | Mean of driest quarter |
| **Rainfall parameters** | Mean of driest month |
|  | Mean of wettest month |
|  | Mean monthly coefficient in variation |
|  | Mean of coolest quarter |
|  | Mean of warmest quarter |
|  | Mean of wettest quarter |
|  | Mean of driest quarter |
