# Supplementary material for: Introduction pathway and climate trump ecology and life history as predictors of establishment success in alien frogs and toads
Source: Ecol Evol. 2012 Jul;2(7):1437–45. doi: 10.1002/ece3.261 (PMC3434934; doi:10.1002/ece3.261)
Supplement: Supplementary file 4 [file ece30002-1437-SD4.doc]

**Table S3. The effect sizes, 95% lower and upper confidence intervals and relative importance of each factor for both data sets.**

| **Variable** | **Coefficient** | **Lower** | **Upper** | **Importance** | **Data Set** |
| --- | --- | --- | --- | --- | --- |
| Climate matching | 1.59 | 0.790 | 2.386 | 1.00 | Excluding BS and CS |
| Intentional | 2.65 | 1.709 | 3.581 | 1.00 | Excluding BS and CS |
| Unknown Intentionality | 1.60 | 0.811 | 2.396 | 1.00 | Excluding BS and CS |
| Unintentional | 1.06 | 0.187 | 1.931 | 1.00 | Excluding BS and CS |
| Distance | -0.99 | -1.806 | -0.176 | 0.93 | Excluding BS and CS |
| Island | 1.13 | 0.152 | 2.118 | 0.87 | Excluding BS and CS |
| Tadpoles | -1.61 | -3.649 | 0.435 | 0.53 | Excluding BS and CS |
| Range size | -1.01 | -2.252 | 0.218 | 0.52 | Excluding BS and CS |
| Congeneric presence | 0.55 | -0.375 | 1.477 | 0.33 | Excluding BS and CS |
| Niche breadth | 0.33 | -0.658 | 1.323 | 0.26 | Excluding BS and CS |
| Climate matching | 1.46 | 0.654 | 2.267 | 1.00 | Including BS and CS |
| Intentional | 2.54 | 1.639 | 3.434 | 1.00 | Including BS and CS |
| Intentional Unknown | 1.56 | 0.797 | 2.331 | 1.00 | Including BS and CS |
| Unintentional | 0.94 | 0.072 | 1.804 | 1.00 | Including BS and CS |
| Distance | -0.98 | -1.822 | -0.151 | 0.93 | Including BS and CS |
| Island | 1.08 | 0.117 | 2.034 | 0.87 | Including BS and CS |
| Tadpoles | -2.27 | -4.510 | -0.030 | 0.63 | Including BS and CS |
| Range size | -1.22 | -2.674 | 0.219 | 0.56 | Including BS and CS |
| Clutch size | 1.00 | -0.696 | 2.713 | 0.32 | Including BS and CS |
| Body size | -0.88 | -2.414 | 0.655 | 0.32 | Including BS and CS |
| Niche breadth | 0.72 | -0.221 | 1.654 | 0.32 | Including BS and CS |
| Congeneric presence | 0.43 | -0.482 | 1.361 | 0.23 | Including BS and CS |
